# Real-Time Estimation of Arterial Partial Pressure of Carbon Dioxide in Patients Undergoing General Anesthesia: Predictive Modeling Study

**DOI:** 10.2196/64855

**Published:** 2025-09-16

**Authors:** Ah Ra Lee, Jun Ho Lee, Sooyoung Yoo, Ho-Young Lee, Hyun Ho Kim

**Affiliations:** 1 Office of eHealth Research and Business, Seoul National University Bundang Hospital, 172, Dolma-ro, Bundang-gu, Seongnam-si, 13605, Republic of Korea; 2 Department of Anesthesiology and Pain Medicine, Jeonbuk National University Medical School and Hospital, Jeonju, Republic of Korea; 3 Department of Nuclear Medicine, Seoul National University Bundang Hospital, Seongnam-si, Republic of Korea; 4 Department of Pediatrics, Jeonbuk National University School of Medicine, 20, Geonji-ro, Deokjin-gu, Jeonju, 54907, Republic of Korea, 82 632501460; 5 Research Institute of Clinical Medicine, Jeonbuk National University-Biomedical Research Institute on Jeonbuk National University Hospital, Jeonju, Republic of Korea

**Keywords:** anesthesia, arterial partial pressure of carbon dioxide, artificial intelligence, AI, blood gas monitoring, end-tidal carbon dioxide

## Abstract

**Background:**

Adequate ventilation in mechanically ventilated patients is contingent upon the monitoring of the arterial partial pressure of carbon dioxide (PaCO_2_) during general anesthesia. Despite its significance, continuous monitoring remains challenging due to the imprecision of noninvasive estimations and the invasive nature of traditional methods such as arterial blood gas analysis.

**Objective:**

This study aimed to develop a machine learning model to continuously estimate PaCO_2_ in mechanically ventilated patients, with the goal of potentially improving intraoperative monitoring accuracy under general anesthesia.

**Methods:**

This retrospective study used the VitalDB dataset from Seoul National University Hospital, comprising records of 6388 noncardiac surgery patients between August 2016 and June 2017. After applying inclusion and exclusion criteria, data from 2304 surgical cases (4651 PaCO_2_ measurement event points) were analyzed. The CatBoost regressor model was trained to predict PaCO_2_ using noninvasive physiological parameters and clinical information. The model’s performance was evaluated using nested cross-validation across hypocapnic (<35 mm Hg), normocapnic (35‐45 mm Hg), and hypercapnic (>45 mm Hg) subgroups and compared to conventional estimation methods based on end-tidal carbon dioxide (ETCO_2_).

**Results:**

The developed model demonstrated superior overall performance compared to traditional estimations. It achieved a mean absolute error of 2.38 mm Hg and an average intraclass correlation coefficient of 0.87. Furthermore, 90.02% of the model’s estimations fell within the clinically highly acceptable range (error<±5 mm Hg) while only 1.20% of errors exceeded ±10 mm Hg. Performance improvements were observed across all PaCO_2_ subgroups.

**Conclusions:**

The developed model provides more accurate and reliable estimates of PaCO_2_ than traditional ETCO_2_-based methods. This approach shows potential for facilitating real-time monitoring and timely clinical interventions. This study demonstrated the potential of artificial intelligence to enhance continuous monitoring of PaCO_2_; however, further validation, including prospective studies assessing clinical impact, is necessary.

## Introduction

Monitoring the arterial partial pressure of carbon dioxide (PaCO_2_) is essential during general anesthesia, as it is a fundamental indicator of respiratory status in mechanically ventilated patients. These patients are unable to breathe on their own because anesthetic drugs and neuromuscular blocking agents suppress their respiratory responses [1]. During mechanical ventilation, the respiratory rate (RR) is carefully adjusted through continuous patient assessments to ensure adequate ventilation [2,3]. PaCO_2_ is a crucial indicator of the equilibrium between the production and elimination of carbon dioxide [4,5]. Abnormal levels of PaCO_2_ may suggest inadequate ventilation, respiratory insufficiency, or compromised cardiovascular function, which could result in unfavorable surgical outcomes.

Despite its importance, achieving continuous monitoring of PaCO_2_ using arterial blood gas analysis (ABGA) has practical limitations. While ABGA remains the gold standard, providing accurate PaCO_2_ measurements along with other vital information, such as pH and pO_2_, and closely tracking PaCO_2_ changes require repeated arterial sampling, even with an indwelling arterial line (A-line). This necessity for repeated invasive procedures carries resource implications for personnel time and consumables, with costs varying across health care systems [6]. Furthermore, frequent sampling while utilizing a procedure with generally low individual risk carries inherent cumulative risks associated with repeated invasive interventions [7,8]. Crucially, the intermittent nature of ABGA may not fully capture rapid physiological fluctuations occurring during dynamic surgical periods, potentially delaying necessary clinical interventions.

End-tidal carbon dioxide (ETCO_2_) is a fundamental component of anesthetic practice, recommended by the American Society of Anesthesiologists (ASA) [9,10], and is commonly used to estimate PaCO_2_. ETCO_2_ reflects the partial pressure of CO2 at the end of exhalation. While often correlated, a gradient typically exists between PaCO_2_ and ETCO_2_, usually ranging from 3 to 5 mm Hg in individuals with normal lung function [11]. However, the accuracy of ETCO_2_ and PaCO_2_ surrogates can be compromised by various physiological and technical factors. Conditions such as ventilation-perfusion mismatch, increased physiological dead space, and significant changes in cardiac output (CO) can widen this gradient and disrupt the correlation between ETCO_2_ and PaCO_2_ [12-14]. Furthermore, patient-specific variables, including underlying pulmonary pathology, hemodynamic status, and metabolic rate, can further complicate the relationship between these two parameters. Prior research has highlighted the variable precision of ETCO_2_ for estimating PaCO_2_, particularly in patients with respiratory disease [15,16]. Studies have also indicated weaker correlations in certain challenging clinical scenarios or patient populations where gas exchange is significantly impaired [17-19]. Therefore, relying solely on ETCO_2_ measurements to guide ventilation management may not always provide the necessary accuracy, especially during periods of rapid physiological change, which are common in surgery.

While a substantial disparity between PaCO_2_ and ETCO_2_ measurements has been previously linked to a higher risk of mortality, developing noninvasive methods for estimating PaCO_2_ more precisely than ETCO_2_ alone remains an ongoing challenge. The difficulty lies in accurately accounting for the simultaneous influence of numerous, interacting physiological factors that affect CO_2_ kinetics. Recent research demonstrates the potential of artificial intelligence (AI) and machine learning (ML) to effectively model intricate biological variables at an individual patient level, potentially overcoming the limitations of simpler estimation approaches [20]. ML-driven prediction models possess the ability to identify and learn complex, nonlinear relationships among multiple input variables (like those readily available from noninvasive intraoperative monitoring), even without prior assumptions about independence or linearity.

Therefore, this study developed an ML-based prediction model to continuously estimate PaCO_2_ using readily available, noninvasive parameters collected during surgical operations. This approach leverages the capability of ML algorithms to process complex, multidimensional data and potentially capture patient-specific variability more effectively than single-parameter estimates like ETCO_2_. The objective of this study was to assess the feasibility of achieving accurate and reliable real-time PaCO_2_ estimation across diverse surgical procedures and patient populations using a large dataset of intraoperative recordings. By providing clinicians with continuous, noninvasive estimates of PaCO_2_, the developed model holds the potential to enhance intraoperative physiological monitoring, complementing standard methods like ABGA and ETCO_2_; facilitate timely adjustments to ventilation; and ultimately contribute to improved patient outcomes following general anesthesia. Through this study, we aim to contribute to the advancement of perioperative medicine by harnessing the power of AI to optimize patient care in the operating room.

## Methods

### Study Design and Settings

This was a retrospective study using VitalDB, an open dataset containing intraoperative biosignal data and perioperative clinical information from Seoul National University Hospital (SNUH), a tertiary-level hospital in South Korea [21]. The dataset encompassed 6388 cases of noncardiac surgery, with an average of 2.8 million data points per case, collected from August 2016 to June 2017. Vital signs were recorded during surgery, while pertinent clinical information was retrospectively retrieved from the electronic medical records system.

### Ethical Considerations

This study utilized a publicly available, deidentified dataset (VitalDB). The collection and use of this dataset for research purposes were approved by the Institutional Review Board (IRB) of SNUH (H-1408-101-605). The requirement for obtaining individual informed consent was waived by the IRB because the dataset contained only deidentified data. All data were anonymized before inclusion in the database, ensuring patient privacy and confidentiality. No compensation was provided to participants as part of this secondary data analysis.

### Case Selection

Case selection was performed based on the following inclusion and exclusion criteria. Surgical cases involving patients meeting the following criteria were eligible: (1) aged 18-80 years, (2) mechanically ventilated under general anesthesia using an oral airway, (3) had ASA physical status classification grade 5 or lower, (4) had ETCO_2_ monitoring records, and (5) had at least 1 or more PaCO_2_ measurement records obtained during surgery. Each PaCO_2_ measurement obtained via ABGA was defined as an event point. PaCO_2_ measurements before or after surgery were excluded and not considered event points.

### Event Point Definition and Timing

**Figure 1.**
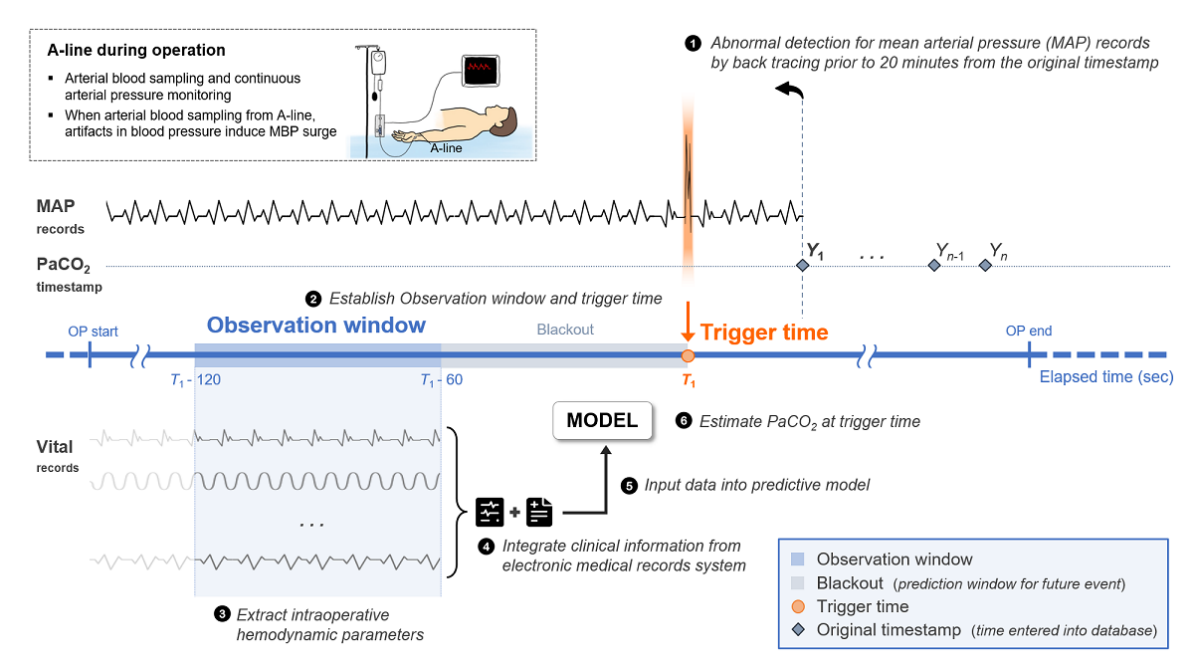
Schematic diagram of the event point definition and feature extraction process for real-time PaCO_2_ estimation. The process includes (1) detection of an MAP surge to refine the ABGA timestamp, defining the trigger time for PaCO_2_ estimation; (2) establishment of a 60-second observation window preceding a blackout period before the trigger time; (3) extraction of medial values from intraoperative biosignals within the observation window; (4) integration of clinical information from electronic medical records; and (5) input of these features into ML-based model to estimate PaCO_2_ at the trigger time. A-line: arterial line; ABGA: arterial blood gas analysis; MAP: mean arterial pressure; MBP: mean blood pressure; ML: machine learning; OP: operation; PaCO_2_: partial pressure of carbon dioxide.

Each event point was defined by a trigger time and an observation window for the prediction model that estimates PaCO_2_ based on noninvasive parameters (Figure 1). The trigger time is defined as the time at which an estimation of PaCO_2_ is performed based on intraoperative biosignals and perioperative clinical information. The observation window is the time frame during which the biosignals were extracted for model training.

PaCO_2_ is measured via ABGA, which evaluates arterial gas concentrations such as carbon dioxide and oxygen. Usually, a small volume of blood is extracted from the radial artery using a syringe and a thin needle. During surgery, the anesthesiologists draw arterial blood via an A-line. The temporal point of the ABGA records might be compromised as a result of the transit time of the specimen to the analyzer or manual data entry delays. The timestamp of ABGA records frequently denotes the moment that analysis results are entered into the database instead of precisely indicating the moment at which blood is drawn from the patient.

To increase the precision of the ABGA timestamp, in this study, we utilized mean arterial pressure (MAP) recordings available in VitalDB. We hypothesized that the ABGA procedure (blood draw via A-line) induces a rapid increase in the MAP due to transient line occlusion or a patient physiological reaction. On the basis of this hypothesis, we employed a *z* score–based outlier detection approach. Abnormal periods surpassing a *z* score of 3 in MAP records were identified by observing the 20-minute interval that preceded the originally recorded ABGA timestamp. The nearest such abnormal period was defined as the estimated actual timepoint at which ABGA was performed. The trigger time for feature extraction was set at 60 seconds prior to the newly calculated ABGA timestamp to capture the patient’s physiological state immediately preceding the likely blood draw event. In this phase, the PaCO_2_ values that did not have identifiable MAP surge points meeting these criteria within the preceding 20-minute window were excluded because they were not considered reliably timed event points. Of the 6311 potentially relevant PaCO_2_ measurements considered for this timestamp adjustment, 720 (approximately 11.4%) were excluded due to the absence of a detectable MAP surge. The median (IQR) difference between the newly estimated ABGA timepoint based on MAP surge and the original database timestamp for the remaining measurements was 34.00 (22.00‐54.00) seconds, with a mean standard deviation (SD) of 47.29 (57.53) seconds (range 0-936.00 seconds), indicating that the original database timestamp often did not precisely reflect the physiological event of blood sampling. While this pressure-surge detection method provides a systematic approach to approximate the blood sampling time based on physiological responses, we acknowledge that its precise accuracy compared to the true sampling time was not formally validated against a gold standard timestamp in this study.

### Data Preparation

A total of 19 variables were selected based on previous literature and domain knowledge. These variables were classified as follows: clinical information, including age, sex, height, weight, surgical approach, surgery type, and preoperative pulmonary function test (PFT) results; and intraoperative biosignals, including body temperature (BT), heart rate, percutaneous oxygen saturation (SpO_2_), minute ventilation from the ventilator (MV), positive end-expiratory pressure (PEEP), peak inspiratory pressure (PIP), plateau pressure (PPLAT), mean airway pressure (MAWP), RR based on capnography, tidal volume (TV), fraction of inspired oxygen (FiO_2_), and ETCO_2_.

During the 60-second observation window before the trigger time, intraoperative biosignals were extracted. Extreme outliers were eliminated using the IQR method, removing data points outside 3 times of the IQR below the first quartile or above the third quartile. Aberrant points deemed theoretically unacceptable were also removed based on clinical expertise. Median values for each biosignal in the observation windows were then calculated and used as features for the prediction model, along with the corresponding surgical patients’ electronic medical records. This approach simplifies the high-frequency biosignal data into static features for point-in-time PaCO_2_ estimation but does not explicitly model temporal dependencies within the observation window or directly track dynamic changes over longer periods.

Furthermore, additional features were established by utilizing feature engineering techniques [20]. The variables for PFT results were reportedly classified into nine classes in the original dataset description; however, due to potential inconsistencies or inadequate representation of minority classes, we regenerated the preoperative PFT as an indicator variable, categorizing it into three grades: obstructive, restrictive, and mixed type [22]. Another feature engineering technique employed in this study involved generating interaction features that comprise combinations of two or more existing variables. The incorporation of interaction features, representing known physiological indices, primarily aimed to improve model interpretability by including recognized clinical parameters, although tree-based models like CatBoost can implicitly capture interactions.

Several interaction features, including TV per kilogram of ideal body weight (TV/IBW), oxygen saturation to FiO2 ratio (SpO_2_/FiO_2_), PEEP to FiO_2_ ratio (PEEP/FiO_2_), compliance of the respiratory system (CRS), and rapid shallow breathing index (RSBI), were generated using domain knowledge. Each of these interaction features brings a dimension of clinical relevance that reflects the interaction between multiple aspects of patient respiratory mechanics and ventilator settings. Additionally, this study utilized APCONET, which is an external application programming interface (API) that is able to estimate CO using arterial pressure waveforms as an input feature [23]. Detailed information, such as the unit of data, recording device, and source of data, for all the selected variables is available in Multimedia Appendix 1.

### Preprocessing

Event points containing missing values in any of the selected predictor variables were removed prior to model training. The listwise deletion approach reduced the number of event points from an initial 5591 (derived from cases meeting inclusion criteria before handling missing data for specific event points) to the final 4651 event points used for analysis. The entire dataset was split into training, validation, and testing sets using the nested cross-validation (7 outer folds, 6 inner folds). This configuration was chosen based on the dataset size and common practices aiming for stable performance estimation [24]. All partitioning procedures were conducted at the surgical case level. For example, all data from a single surgical case belonged exclusively to either the training/validation set or the testing set within a given outer fold. This approach was designed to maintain similar data distributions across subsets while ensuring patient independence between training and testing.

Categorical variables were initially processed using OneHotEncoder for compatibility with some preliminary models tested. Continuous variables were scaled using RobustScaler, which scales data using IQR, making it robust to outliers.

### Model Training

To identify a suitable regression model for this task, several ML algorithms were evaluated in preliminary experiments (see Multimedia Appendix 2). Based on its superior performance in these experiments, CatBoostRegressor was selected for final model development and evaluation. The CatBoost model is a robust and effective library for gradient boosting on decision trees. It is particularly adept at handling categorical features natively using its built-in encoder, which was employed in this study for the CatBoost model, thus not requiring the prior OneHotEncoding for these features when training the final CatBoost model [25].

To develop the final predictive model for PaCO_2_, training sets were utilized for training the CatBoostRegressor model, and the corresponding validation set was used for hyperparameter tuning within each outer fold. The training sets were used to train the CatBoostRegressor model, and the validation set was utilized to tune the hyperparameters (see Multimedia Appendix 3). During hyperparameter optimization, we utilized Optuna, a framework designed to streamline the hyperparameter tuning process [26]. This framework facilitates the search for the optimal hyperparameter space configuration for a given model in an efficient manner. Subsequently, the performance of the final model was evaluated using held-out testing sets from the seven outer-fold cross-validation methods on the testing sets.

### Data Analysis

In this study, estimating PaCO_2_ based on noninvasive parameters was approached as a regression predictive modeling task. To assess the effectiveness of the ML-based model, we conducted a comparison with two baseline methods that employed the ETCO_2_ value. One method was a simple offset model (ETCO_2_+5 mm Hg), while the other method involved utilizing linear regression with ETCO_2_ measurements as the sole predictor.

Model performance was assessed using two commonly employed metrics: mean absolute error (MAE) and root mean squared error (RMSE). Additionally, we evaluated model performance across different conditions by establishing subgroups. The subgroups were established based on PaCO_2_ levels as follows: hypocapnic (<35 mm Hg), normocapnic (35-45 mm Hg), and hypercapnic (>45 mm Hg) cases. A Bland-Altman plot was used to calculate the limits of agreement and analyze the agreement between the real and predictive values; additionally, the intraclass correlation coefficient (ICC) was utilized to evaluate the relative reliability and consistency of the model compared to actual measurements [27,28].

To assess the clinical utility of the predictive model, the percentage of estimation errors for PaCO_2_ was computed by calculating the differences between the real and predicted values. Disparities below 5 mm Hg were deemed highly acceptable, those between 5 and 10 mm Hg were moderately acceptable, and any value exceeding 10 mm Hg was considered unacceptable. The establishment of this threshold was based on prior research and Clinical Laboratory Improvement Amendments recommendations [29].

Furthermore, model interpretability was analyzed using the Shapley additive explanation (SHAP) value [30]. The SHAP values, derived from an additive feature attribution model, succinctly illustrate the impact of the input variables on the model outputs, enhancing the understanding of the model’s decision-making process.

The performance metrics were evaluated by averaging the results from the testing sets across the outer folds and computing a CI of 95%. All experimental and data analysis procedures were conducted in the Python 3.10.12 environment.

## Results

A total of 2304 surgical cases (with 4651 event points) were eligible and analyzed to develop and validate the proposed prediction model. The statistical summaries for PaCO_2_ and ETCO_2_ measurements for the included event points are displayed in Table 1.

**Table 1.** Statistical summaries for PaCO_2_^a^ and ETCO_2_^b^ measurements.

Characteristic	Total (n=4651)	Subgroups		
		Hypocapnic (n=179)	Normocapnic (n=3328)	Hypercapnic (n=1144)
PaCO_2_ (mm Hg), median (IQR)	42.00 (39.00‐45.00)	34.00 (33.00‐34.00)	41.00 (38.00‐43.00)	48.00 (47.00‐51.00)
ETCO_2_ (mm Hg), median (IQR)	35.00 (33.00‐37.00)	32.00 (30.00‐33.00)	34.00 (33.00‐36.00)	37.00 (35.00‐40.00)
PaCO_2_-ETCO_2_ (mm Hg difference), median (IQR)	7.00 (5.00‐10.00)	2.00 (0.50‐3.00)	6.00 (4.00‐8.00)	12.00 (9.00‐14.00)

a PaCO_2_: partial pressure of carbon dioxide.

b ETCO_2_: end-tidal carbon dioxide.

The mean values in this cohort were 42.52 mm Hg for PaCO_2_ and 34.95 mm Hg for ETCO_2_. The observed differences between PaCO_2_ and ETCO_2_ measurements were often greater than the gap derived from existing knowledge, which was 3-5 mm Hg for healthy individuals, with an average difference of 7.57 mm Hg and a wide range from 16 to 34 mm Hg in our study population. According to the analysis of subgroups categorized by PaCO_2_ levels, 71.55% (n=3328) of all cases used in this study included PaCO_2_ values in the normal range (between 35 and 45 mm Hg), whereas 3.85% of the cases (n=179) were hypocapnic (<35 mm Hg) and 24.60% (n=1144) were hypercapnic (>45 mm Hg). The differences in ETCO_2_ values across subgroups were less pronounced than the differences in PaCO_2_ values, whereas the discrepancy between PaCO_2_ and ETCO_2_ measurements tended to be greater in subgroups with higher PaCO_2_ levels. More detailed descriptive statistics for all variables are provided in Multimedia Appendix 4.

The performance evaluation results of the developed model in comparison to the baseline methods are displayed in Table 2. As shown in Table 2, the error metrics of the baseline method (Baseline 1), which adds 5 mm Hg to ETCO_2_, significantly increased as the subgroup transitions from normocapnic to hypercapnic. High error rates in the hypercapnic group indicate that it is difficult to accurately estimate PaCO_2_ using this baseline method in patients with relatively higher PaCO_2_ values. The performance of the second baseline method (Baseline 2), which is based upon linear regression, exhibited slightly better performance overall than the first one but performed poorly in the hypocapnic group compared to the normocapnic group. In both of the two baseline methods, extreme groups (hypocapnic and hypercapnic cases) result in wider CIs, which suggests that the performance of these methods is less certain in such ranges.

**Table 2.** Performance evaluation results.

Model		MAE^a^ (95% CI)	MSE^b^ (95% CI)	RMSE^c^ (95% CI)
Baseline 1: ETCO_2_^d^+5 mm Hg				
	All	3.64 (3.51-3.77)	24.57 (22.86-26.29)	4.95 (4.78-5.13)
	Hypocapnic	3.64 (2.99-4.28)	19.82 (10.61-29.03)	4.34 (3.33-5.34)
	Normocapnic	2.45 (2.30-2.61)	9.96 (8.86-11.06)	3.15 (2.98-3.32)
	Hypercapnic	7.09 (6.90-7.28)	67.88 (63.73-72.04)	8.24 (7.98-8.49)
Baseline 2: Linear regression				
	All	3.26 (3.17-3.36)	18.70 (17.34-20.05)	4.32 (4.16-4.48)
	Hypocapnic	6.34 (5.62-7.06)	50.20 (32.67-67.73)	6.98 (5.78-8.19)
	Normocapnic	2.52 (2.42-2.62)	9.85 (9.00-10.70)	3.14 (3.00-3.27)
	Hypercapnic	4.93 (4.77-5.10)	39.55 (36.27-42.82)	6.28 (6.02-6.54)
ML-based^e^ model: CatBoost regressor				
	All	2.38 (2.34-2.41)	10.63 (10.13-11.13)	3.26 (3.18-3.34)
	Hypocapnic	3.66 (2.96-4.35)	21.49 (10.23-32.75)	4.51 (3.47-5.56)
	Normocapnic	1.88 (1.81-1.95)	5.81 (5.29-6.33)	2.41 (2.3-2.52)
	Hypercapnic	3.63 (3.50-3.76)	23.05 (21.18-24.91)	4.80 (4.60-4.99)

a MAE: mean absolute error.

b MSE: mean squared error.

c RMSE: root mean squared error.

d ETCO_2_: end-tidal carbon dioxide.

e ML: machine learning.

In contrast, the ML-based model developed in this study exhibited superior performance in all subgroups compared to the other 2 baseline methods, with the normocapnic group achieving particularly noteworthy results. With the ML-based model, the error metrics were MAE of 1.88 (95% CI 1.81‐1.95), mean squared error (MSE) of 5.81 (95% CI 5.29‐6.33), and RMSE of 2.41 (95% CI 2.30‐2.52) in the normocapnic group. For extreme PaCO_2_ values of the hypocapnic and hypercapnic groups, the developed model exhibited MAEs of 3.66 (95% CI 2.96‐4.35) and 3.63 (95% CI 3.50‐3.76), respectively, which were considerably better than the baseline methods in these challenging subgroups. Given that the differences between PaCO_2_ and ETCO_2_ in healthy individuals are known to range from 3 to 5 mm Hg, these results indicate that the ML-based model provides more accurate and reliable results, as evidenced by the consistently lower MAE, MSE, and RMSE values across all subgroups.

**Figure 2.**
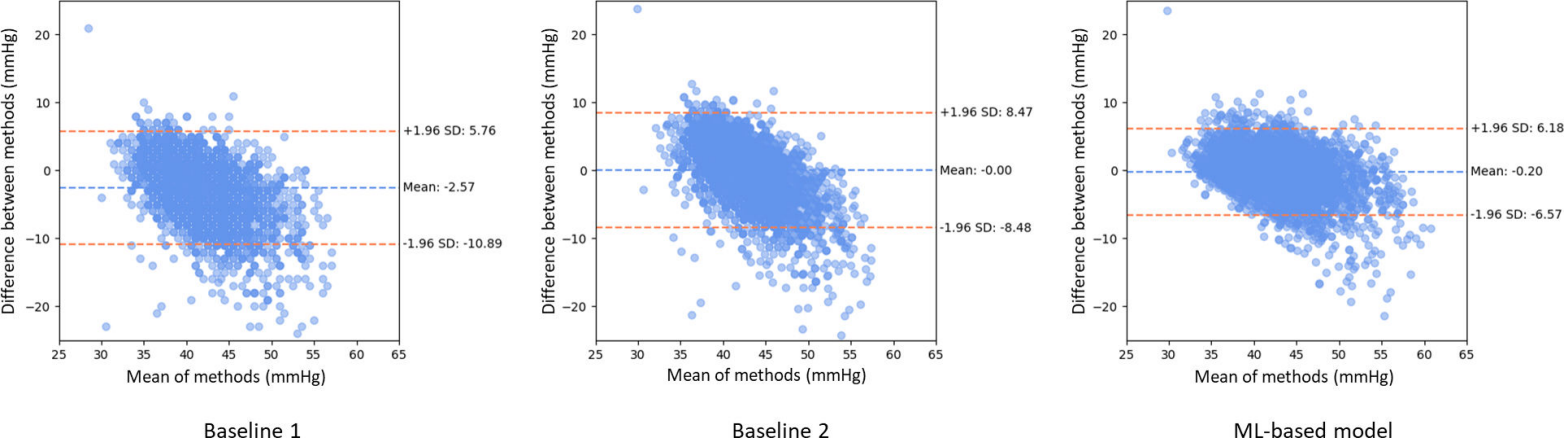
Bland-Altman plots illustrating the agreement between actual PaCO_2_ and estimated PaCO_2_ values by (1) Baseline 1: ETCO_2_+5 mm Hg; (2) Baseline 2: linear regression with ETCO_2_; and (3) the machine learning–based model. The x-axis represents the mean of actual and predicted PaCO_2_ values. The central dashed blue line indicates the mean differences (bias), and the outer dashed orange lines represent the 95% limits of agreement (mean difference ±1.96 SD of the differences). ETCO_2_: end-tidal carbon dioxide; PaCO_2_: partial pressure of carbon dioxide.

Bland-Altman plots comparing the predictive model with the two baseline methods are displayed in Figure 2. The mean difference, indicated by the central blue dashed line, represents the average discrepancy between the predicted and actual PaCO_2_ values. For the second baseline method and the ML-based model, this difference was exceedingly close to 0, suggesting minimal systematic deviation on average, while the first baseline method (ETCO_2_+5 mm Hg) showed a slightly larger negative bias (−2.57 mm Hg). The limits of agreement (±1.96 SD of the differences) with the 95% CIs for the baseline methods ranged from −10.89 to +5.76 and −8.48 to 8.47, respectively, and were narrower for the ML-based model at −6.57 to +6.18. The wider limits of agreement in the baseline models suggest that there is more variability in the differences between the actual and predicted values when estimating the PaCO_2_ with these traditional approaches.

In addition, the Bland-Altman plot for the ML-based model demonstrated that the majority of data points were tightly clustered around the mean difference with less dispersion compared to the baseline methods. While the baseline methods showed some tendency for larger underestimation at higher PaCO_2_ values (negative slope suggestion), the ML-based model exhibited more consistent agreement across the range of PaCO_2_ values, with no evident trend of increasing or decreasing differences correlating with the average PaCO_2_ values. A few outliers were observed, particularly at higher mean values across all methods, suggesting potential specific variability under certain conditions or limitations in the developed model’s performance at extremes.

Table 3 presents the ICC analysis results for the baseline methods and the ML-based model. A statistically significant ICC (*P*<.001) indicates some degree of reliability beyond chance. The narrow 95% CI of the ICC underscores a high degree of confidence in these reliability estimates. An ICC of 0.87 (95% CI 0.86‐0.87) in the ML-based model signified good agreement between predicted and actual values, indicating that the predicted values closely align with the actual values relative to the overall variance. Conversely, the baseline methods yielded ICCs of 0.70 (95% CI 0.68‐0.71) and 0.67 (95% CI 0.65‐0.69), respectively, reflecting a moderate level of agreement.

**Table 3.** The ICC^a^ values between predicted and actual PaCO_2_^b^.

Model	ICC (95% CI)	*P* value
Baseline 1	0.70 (0.68-0.71)	<.001
Baseline 2	0.67 (0.65-0.69)	<.001
ML^c^-based model	0.87 (0.86-0.87)	<.001

a ICC: intraclass correlation coefficient.

b PaCO_2_: partial pressure of carbon dioxide.

c ML: machine learning.

The evaluation results of the clinical utility are displayed in Table 4 as a percentage of the estimation error for the PaCO_2_. The ML-based model exhibited superior performance, with 90.02% of the test set having errors of less than ±5 mm Hg, in contrast to the baseline methods that had exhibited 72.41% and 80.43%, respectively. This represents a substantial absolute increase of nearly 10 percentage points in highly accurate predictions compared to the better baseline. Additionally, for the ML model, errors falling within ±10 mm Hg accounted for 98.80% of the test set. The errors exceeded ±10 mm Hg in only 1.20% of cases. The baseline methods achieved a moderate level of acceptable performance; however, the percentage of errors exceeding ±10 mm Hg was more than double that of the ML-based model. This indicates that the ML-based model demonstrated highly acceptable performance in aspects of clinical utility evaluation.

**Table 4.** Clinical utility evaluation results.

Model	Absolute value of errors (%)		
	<5 mm Hg	5‐10 mm Hg	>10 mm Hg
Baseline 1	72.41	22.70	4.88
Baseline 2	80.43	16.92	2.64
ML^a^-based model	90.02	8.77	1.20

a ML: machine learning.

**Figure 3.**
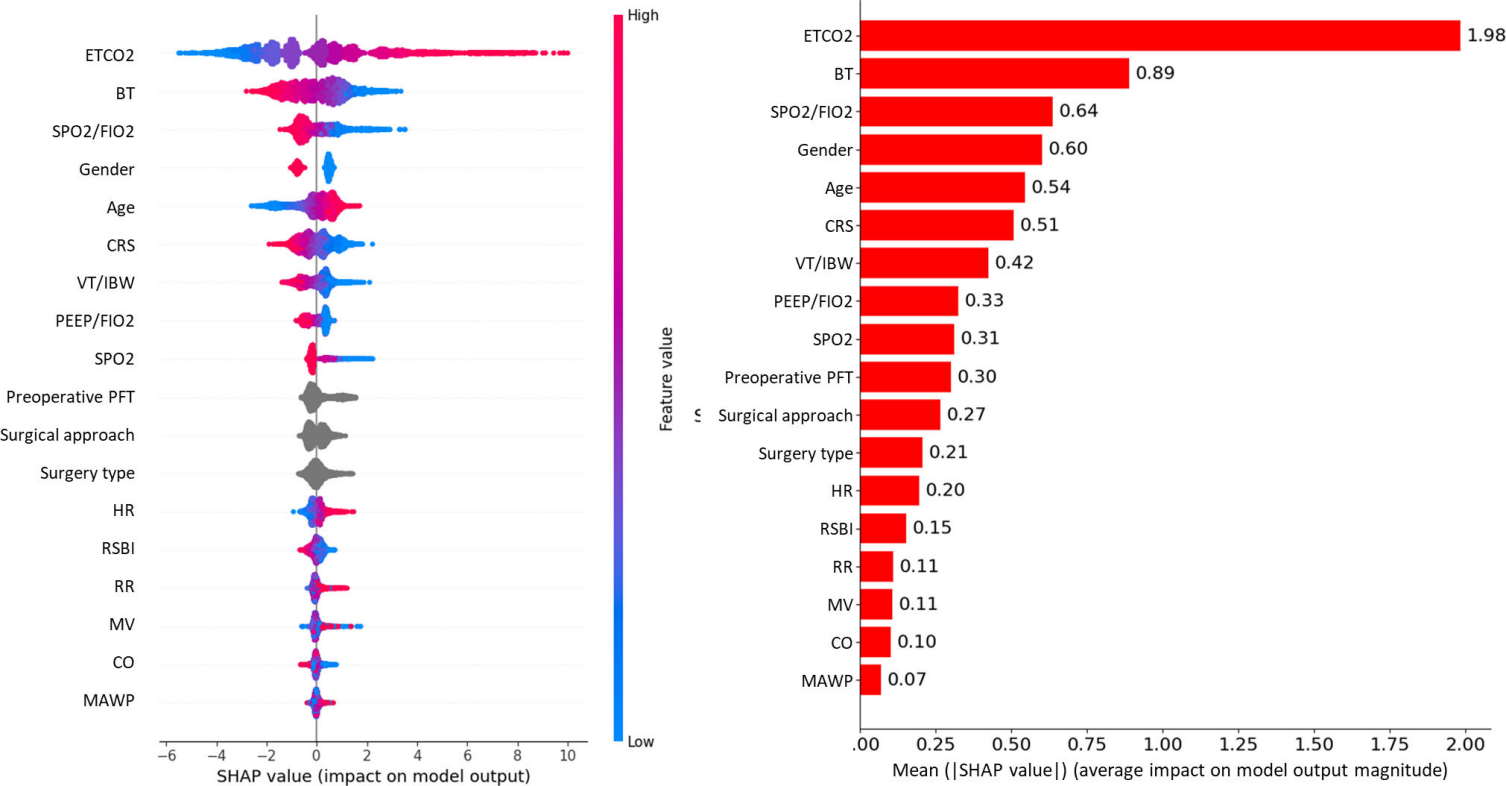
SHAP plots illustrating feature importance for ML-based PaCO_2_ estimation model. Left: SHAP summary plot, where each point represents a Shapley value for a feature and an instance. The position on the y-axis indicates the feature, the position on the x-axis indicates the SHAP value (impact on model output), and the color indicates the feature values (red for high, blue for low). Right: Bar chart of the mean absolute SHAP values, indicating the global importance of each feature in the model. BT: body temperature; CO: cardiac output; CRS: compliance of the respiratory system; ETCO_2_: end-tidal carbon dioxide; FIO_2_: fraction of inspired oxygen; HR: heart rate; IBW: ideal body weight; MAWP: mean airway pressure; ML: machine learning; MV: minute ventilation from the ventilator; PaCO_2_: partial pressure of carbon dioxide; PEEP: positive end-expiratory pressure; PFT: pulmonary function test; RR: respiratory rate; RSBI: rapid shallow breathing index; SHAP: Shapley additive explanation; SPO_2_: percutaneous oxygen saturation; VT: tidal volume.

The feature importance of the ML-based model was analyzed using the SHAP method, as illustrated in Figure 3. The most significant variable was ETCO_2_, as expected, given its direct physiological link to PaCO_2_. Beyond ETCO_2_’s dominant influence, other important features included BT, SpO_2_/FiO_2_, sex, age, and CRS. The SHAP plot suggested that the model output (predicted PaCO_2_ value) tended to be higher with lower BT, SpO_2_/FiO_2_, and CRS, as well as higher age and ETCO_2_. These findings suggest the additional features help refine the PaCO_2_ estimate beyond the baseline provided by ETCO_2_, potentially capturing patient-specific physiological states. Variables such as MAWP, CO, and MV exhibited relatively lower SHAP values with a mix of positive and negative contributions, indicating a minor or context-dependent impact on model decision-making in this analysis.

## Discussion

### Principal Findings

This study developed an ML-based model capable of estimating PaCO_2_ in mechanically ventilated patients under general anesthesia with greater accuracy than traditional ETCO_2_-based methods. Utilizing noninvasive physiological parameters and clinical information, the CatBoost model demonstrated strong overall performance, achieving an MAE of 2.38 mm Hg, an RMSE of 3.26 mm Hg, and an ICC of 0.87, indicating excellent agreement with arterial measurements. Critically, the model significantly increased the proportion of clinically highly acceptable predictions (error<±5 mm Hg) to 90.02%, comparable to 80.43% for a linear regression baseline, and reduced unacceptable errors (>±10 mm Hg) to 1.20% from 2.64%. The model’s superiority was consistent across hypocapnic, normocapnic, and hypercapnic subgroups.

Interpretability analysis using SHAP identified ETCO_2_ as the most influential feature, as anticipated. Beyond the dominant contribution of ETCO_2_, other parameters such as BT, SpO_2_/FiO_2_ ratio, age, sex, and CRS were found to be important for refining PaCO_2_ estimations. For instance, the model tended to predict higher PaCO_2_ values with lower BT, lower SpO_2_/FiO_2_, lower CRS, and higher age, suggesting it learned complex physiological relationships. These findings highlight the value of a multiparameter approach to capture variability not explained by ETCO_2_ alone.

### Comparison to Prior Work

The limitations of relying solely on ETCO_2_ for PaCO_2_ estimation are well documented. While ETCO_2_ provides some insights, the PaCO_2_-ETCO_2_ gradient is known to be variable and influenced by numerous physiological factors, often exceeding the commonly cited 3-5 mm Hg range in healthy individuals [11,16,31]. Our study corroborates this, finding a median gradient of 7 mm Hg (average 7.57 mm Hg, range −16 to 34 mm Hg), underscoring the unreliability of a fixed gradient assumption. Conventional statistical models, such as multivariable linear regression, offer some improvement but are often constrained by linearity assumptions and may not fully capture the complex, nonlinear interactions inherent in physiological systems [32,33].

In contrast, our ML-based approach effectively models these intricate associations by integrating a wider array of biosignals and clinical data. The ability of ML to learn from these complex patterns without a priori assumptions about relationships has shown promise in various medical prediction tasks [20]. Previous studies have also indicated that factors like surgical techniques and patient positioning can affect the PaCO_2_-ETCO_2_ gradient [34], further supporting the need for adaptive models like the one developed in this study, which can account for such patient-specific and contextual variability more effectively than simpler methods.

### Strengths of the Study

This study possesses several strengths that enhance the credibility and potential impact of its findings. First, the use of VitalDB, a large, publicly available, real-world dataset from a tertiary university hospital, provides a diverse cohort from various noncardiac surgeries, improving the generalizability of our results. Second, model performance was rigorously assessed using nested cross-validation, offering a robust estimate of its predictive capabilities on unseen data. Third, the ML model was benchmarked against two clinically relevant baseline methods, clearly demonstrating its superior accuracy. Fourth, our evaluation encompassed not only standard error metrics (MAE, RMSE) but also reliability (ICC) and clinical utility based on predefined error categories (Table 4), providing a multifaceted view of performance. Fifth, the inclusion of SHAP analysis offers a degree of transparency into the model’s decision-making process, which is crucial for clinical translation. Finally, the exploration of an MAP-based timestamping method, while requiring further validation, represents a novel attempt to address a common challenge in retrospective EMR-based research.

### Limitations

Nevertheless, several limitations should be acknowledged when interpreting the results of this study. First, while the ML model outperformed baselines across all PaCO_2_ subgroups, its performance was relatively lower in the hypocapnic and hypercapnic groups compared to the normocapnic group. This may be partly due to data imbalance, as these extreme ranges were less frequently observed. Future work could explore techniques like targeted data augmentation or specialized modeling to address this. Second, our SHAP analysis focused on explaining direct PaCO_2_ predictions. As ETCO_2_ is inherently a dominant feature, this makes it harder to isolate the specific contributions of other features to the PaCO_2_—ETCO_2_ gradient. Analyzing this gradient directly would be a valuable future direction. Third, the model relies on point-in-time estimations using median values from a 60-second window, simplifying the rich time-series data available. This approach does not capture temporal trends or predict rapid PaCO_2_ changes. Fourth, the MAP-surge-based ABGA timestamping method, though systematically applied, was not formally validated against a gold-standard timing reference. Any imprecision here could introduce noise into the feature-target alignment. Fifth, the listwise deletion approach for handling missing data, which reduced our event points from 5951 to 4651, may have introduced selection bias if the pattern of missingness was not completely at random and reduced the overall sample size available for training. Seventh, estimated CO via an external API did not emerge as a highly influential feature in SHAP plots. This might be attributed to the indirect nature or potential inaccuracies of the CO estimation rather than CO itself lacking physiological relevance. Finally, being a single-institution study, the findings require external validation to ensure generalizability across different settings and populations.

### Future Directions

Building on these findings and limitations, several avenues for future research are essential for advancing noninvasive PaCO_2_ monitoring. First and foremost, external validation of the ML model in diverse, multicenter clinical settings is crucial to confirm its robustness and general applicability. Second, developing time-series models, such as recurrent neural networks, long short-term memory, and transformers, which can process the continuous stream of biosignals, is a key next step. This could improve accuracy and enable the prediction of PaCO_2_ trends and rapid changes. Third, future studies should explicitly investigate the model’s capacity to track longitudinal changes in the PaCO_2_-ETCO_2_ gradient within individual patients. Exploring the linkage between the predicted PaCO_2_-ETCO_2_ gradient and critical events like hemodynamic instability could yield clinical value. Fourth, research correlating intraoperative PaCO_2_ deviations identified by accurate monitoring with postoperative outcomes would further strengthen the rationale for enhanced continuous monitoring. Finally, the MAP-based timestamping approach warrants further investigation and validation.

### Conclusions

This study demonstrated that an ML-based model integrating multiple noninvasive parameters can estimate PaCO_2_ with higher accuracy and reliability than traditional ETCO_2_-based methods in mechanically ventilated surgical patients. The model shows particular strength in increasing the proportion of highly accurate predictions. While acknowledging the need for further development, particularly in incorporating time-series data and external validation, this work highlights the considerable potential of AI to serve as a valuable supplementary tool for enhancing respiratory monitoring and patient management in the perioperative setting.

## Supplementary material

10.2196/64855 Multimedia Appendix 1 List of selected variables.

10.2196/64855 Multimedia Appendix 2 Preliminary experiment results for a performance comparison of machine learning algorithms.

10.2196/64855 Multimedia Appendix 3 Hyperparameter optimization with nested cross-validation.

10.2196/64855 Multimedia Appendix 4 Descriptive statistics of the selected variables.

## Abbreviations

A-line: arterial line
ABGA: arterial blood gas analysis
AI: artificial intelligence
API: application programming interface
ASA: American Society of Anesthesiologists
BT: body temperature
CO: cardiac output
CRS: compliance of the respiratory system
ETCO_2_: end-tidal carbon dioxide
FIO_2_: fraction of inspired oxygen
IBW: ideal body weight
ICC: intraclass correlation coefficient
IRB: Institutional Review Board
MAE: mean absolute error
MAP: mean arterial pressure
MAWP: mean airway pressure
ML: machine learning
MSE: mean squared error
MV: minute ventilation from the ventilator
PaCO_2_: partial pressure of carbon dioxide
PEEP: positive end-expiratory pressure
PFT: pulmonary function test
PIP: peak inspiratory pressure
PPLAT: plateau pressure
RMSE: root mean squared error
RR: respiratory rate
RSBI: rapid shallow breathing index
SHAP: Shapley additive explanation
SNUH: Seoul National University Hospital
SPO2: percutaneous oxygen saturation
TV: tidal volume
