# Supplementary material for: Real-Time Estimation of Arterial Partial Pressure of Carbon Dioxide in Patients Undergoing General Anesthesia: Predictive Modeling Study
Source: JMIR Med Inform. 2025 Sep 16;13:e64855. doi: 10.2196/64855 (PMC12439857; doi:10.2196/64855)
Supplement: Multimedia Appendix 1 [file medinform-v13-e64855-s001.pdf]

**Multimedia appendix 1.** List of selected variables

| Features | Description | Unit | Source | Recording device (Company) | Acquisition interval (sec) |
|---|---|---|---|---|---|
| **Demographics** | | | | | |
| Age | Age | years | EMR | - | - |
| Gender | Gender (men \| women) | - | EMR | - | - |
| Height* | Height | ㎝ | EMR | - | - |
| Weight* | Weight | kg | EMR | - | - |
| **Clinical information** | | | | | |
| Approach | Surgical approach (open \| videoscopic \| robotic) | - | EMR | - | - |
| Optype | Surgery type (major resection \| minor resection \| transplantation \| stomach \| hepatic \| biliary/pancreas \| colorectal \| vascular \| thyroid \| breast \| others) | - | EMR | - | - |
| PFT | Preoperative pulmonary function test (normal \| mild obstructive \| mild restrictive \| mixed or pure obstructive \| moderate obstructive \| moderate restrictive \| severe restrictive \| borderline obstructive \| severe obstructive) | - | EMR | - | - |
| **Hemodynamic parameters** | | | | | |
| BT | Body temperature | ℃ | Patient monitor | Solar8000M (GE healthcare) | 2 |
| HR | Heart rate | /min | Patient monitor | Solar8000M (GE healthcare) | 2 |
| $SPO_2$ | Percutaneous oxygen saturation | % | Patient monitor | Solar8000M (GE healthcare) | 2 |
| $ETCO_2$ | End-tidal carbon dioxide | ㎜Hg | Patient monitor | Solar8000M (GE healthcare) | 2 |
| MV | Minute ventilation (from ventilator) | L/min | Patient monitor | Solar8000M (GE healthcare) | 2 |
| MAWP | Mean airway pressure | mbar | Anesthesia machine | Primus (Drager) | 7 |
| RR | Respiratory rate based on capnography | /min | Anesthesia machine | Primus (Drager) | 7 |
| PEEP* | Positive and expiratory pressure | mbar | Anesthesia machine | Primus (Drager) | 7 |
| PIP* | Peak inspiratory pressure | mbar | Anesthesia machine | Primus (Drager) | 7 |
| PPLAT* | Plateau pressure | mbar | Anesthesia machine | Primus (Drager) | 7 |
| VT* | Tidal volume | mL | Anesthesia machine | Primus (Drager) | 7 |
| $FIO_2$* | Fraction of inspired oxygen | % | Anesthesia machine | Primus (Drager) | 7 |
| **Constructed Features** | | | | | |

| | | | | | |
|---|---|---|---|---|---|
| CO | Estimated cardiac output | L/min | External API | - | 1/50 |
| VT/IBW | Tidal volume per kilogram of ideal body weight | - | Gender; Height; VT | - | - |
| $SPO_2/FIO_2$ | Oxygen saturation to fraction of inspired oxygen | - | $SPO_2$; $FIO_2$ | - | - |
| $PEEP/FIO_2$ | Positive end expiratory pressure to fraction of inspired oxygen | - | PEEP; $FIO_2$ | - | - |
| CRS | Compliance of the respiratory system | - | VT; PEEP; PPLAT | - | - |
| RSBI | Rapid shallow breathing index | - | VT; RR | - | - |

* Used for establishing constructed features (not for model training)
