# Supplementary material for: Real-Time Estimation of Arterial Partial Pressure of Carbon Dioxide in Patients Undergoing General Anesthesia: Predictive Modeling Study
Source: JMIR Med Inform. 2025 Sep 16;13:e64855. doi: 10.2196/64855 (PMC12439857; doi:10.2196/64855)
Supplement: Multimedia Appendix 2 [file medinform-v13-e64855-s002.pdf]

Multimedia appendix 2. Preliminary experiment results for a performance comparison of machine learning algorithms

Model	MAE (95% CI)	MSE (95% CI)	RMSE (95% CI)
**Univariate regression ($ETCO_2$)**			
$ETCO_2$ + 3 mmHg	4.88 [4.71, 5.06]	38.81 [36.41, 41.20]	6.23 [6.03, 6.42]
$ETCO_2$ + 5 mmHg	3.64 [3.51, 3.77]	24.57 [22.86, 26.29]	4.95 [4.78, 5.13]
LinearRegression	3.26 [3.17, 3.36]	18.70 [17.34, 20.05]	4.32 [4.16, 4.48]
**Multivariable regression**			
LinearRegression	2.63 [2.55, 2.70]	12.84 [11.92, 13.75]	3.58 [3.45, 3.71]
LogisticRegression	2.81 [2.74, 2.88]	14.78 [14.08, 15.48]	3.84 [3.75, 3.93]
ExtaTreeRegressor	3.80 [3.61, 3.99]	25.63 [23.04, 28.22]	5.06 [4.80, 5.32]
DecisionTreeRegressor	3.69 [3.59, 3.80]	24.59 [22.79, 26.38]	4.96 [4.77, 5.14]
AdaBoostRegressor	3.10 [3.00, 3.20]	15.61 [14.7, 16.52]	3.95 [3.83, 4.07]
ExtraTreesRegressor	2.53 [2.44, 2.61]	11.73 [11.00, 12.45]	3.42 [3.32, 3.53]
BaggingRegressor	2.69 [2.60, 2.77]	13.15 [12.30, 14.00]	3.62 [3.51, 3.74]
GradientBoostingRegressor	2.42 [2.35, 2.49]	10.96 [10.32, 11.59]	3.31 [3.21, 3.40]
RandomForestRegressor	2.54 [2.45, 2.63]	11.89 [11.11, 12.68]	3.45 [3.33, 3.56]
HistGradientBoosting	2.45 [2.37, 2.53]	11.13 [10.53, 11.73]	3.33 [3.24, 3.43]
XGBRegressor	2.62 [2.54, 2.70]	12.44 [11.83, 13.05]	3.53 [3.44, 3.61]
LGBMRegressor	2.45 [2.38, 2.52]	11.07 [10.48, 11.65]	3.33 [3.24, 3.41]
CatBoostRegressor	2.39 [2.34, 2.43]	10.78 [10.23, 11.34]	3.28 [3.20, 3.37]

$ETCO_2$=End-tidal carbon oxide; MAE=Mean absolute error; MSE=Mean squared error; RMSE=Root mean squared error; CI=Confidence interval; XGB=Extreme gradient boosting; LGBM: Light gradient boosting machine
