# Supplementary material for: Real-Time Estimation of Arterial Partial Pressure of Carbon Dioxide in Patients Undergoing General Anesthesia: Predictive Modeling Study
Source: JMIR Med Inform. 2025 Sep 16;13:e64855. doi: 10.2196/64855 (PMC12439857; doi:10.2196/64855)
Supplement: Multimedia Appendix 3 [file medinform-v13-e64855-s003.pdf]

Multimedia appendix 3. Hyperparameter optimization with nested cross-validation

Nested cross-validation (CV) is a techniques that employs cross-validation in both an outer loop and an inner loop [1]. This method identifies the optimal hyperparameters and provides an unbiased assessment of the model's performance.

In this study, the entire dataset is partitioned into seven folds. The testing set is designated as one fold during each iteration of the outer loop, while the training set is composed of the remaining folds. The remaining folds serve as the training set for that iteration, while the outer loop sequentially uses each fold as a testing set. The training set is further divided into separate training and validation sets during each iteration of the outer loop to optimize hyperparameters. The validation set for each inner fold is utilized precisely once to determine the hyperparameters that maximize the model's performance on the inner validation set.

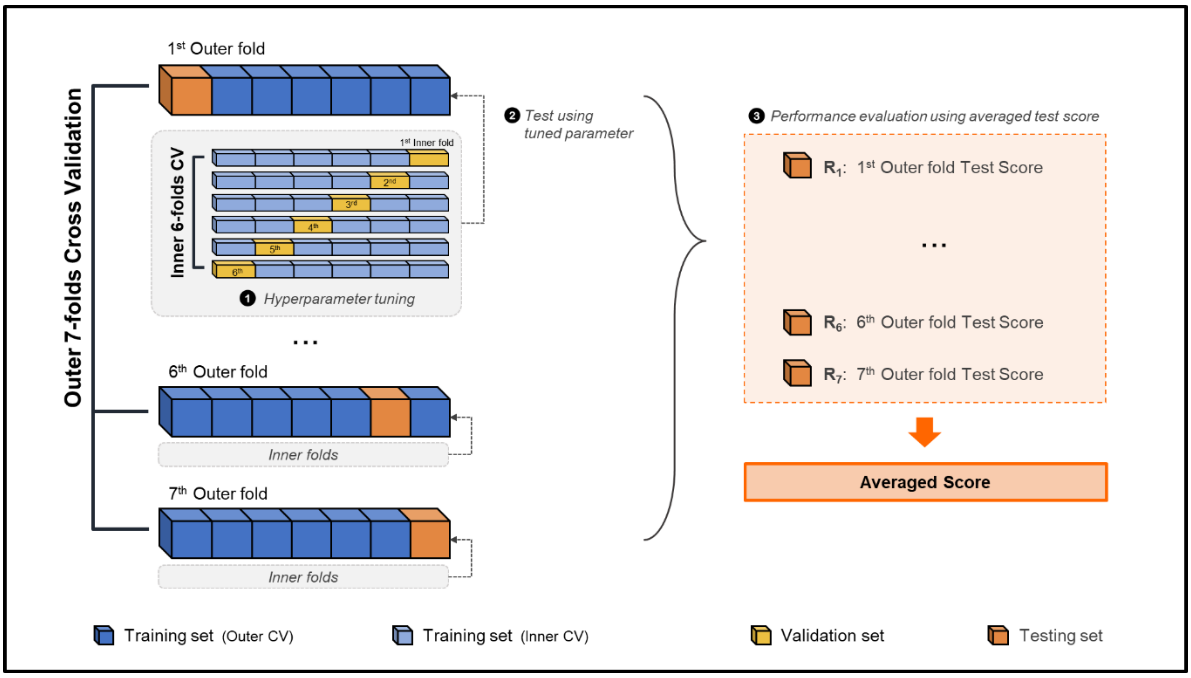

Supplementary Figure. An overview of nested cross-validation for hyperparameter tuning and model evaluation

Hyperparameter tuning is performed using the Optuna framework, which simplifies the hyperparameter optimization process [2]. This study utilized Bayesian optimization, a robust strategy for hyperparameter tuning [3]. Bayesian optimization establishes a probabilistic model of the function by associating hyperparameter values with the target metric. Unlike grid search or random search, Bayesian optimization uses prior outcomes to inform the hyperparameter selection process, aiming to identify the optimal hyperparameters more efficiently. The tree-structured Parzen estimator (TPE) sampler was employed in this study due to its efficacy in rapidly finding optimal hyperparameters [4]. Additionally, the TPE sampler is a capable of handling a wide range of hyperparameters, such as continuous, discrete, and categorical variables. This study used the CatBoost model [5], [6] and the hyperparameters used for the optimization process are listed in the Supplementary Table.

Supplementary Table. Search space for hyperparameter optimization

Parameter	Description	Type	Range
depth	It refers to the depth of the tree, which affects how complex the learned models are.	Integer	4 to 8
learning_rate	It controls the speed at which the model learns by scaling the contribution of each individual tree when it is added to the ensemble.	Float (log-uniform distribution)	0.001 to 0.03
l2_leaf_reg	It configures the L2 regularization term on the weight of leaves.	Float (log-uniform distribution)	0.001 to 10.0
random_strength	It adds noise to the input variables, which helps to avoid overfitting.	Integer	1 to 100
border_count	The number of splits for numerical variables.	Integer	50 to 255
bagging_temperature	It controls the intensity of Bayesian bagging.	Float	1.0 to 10.0

After the inner folds are used to identify the optimal hyperparameters, the model is trained on the outer loop's training set using these hyperparameters. The test set of the outer loop is subsequently employed to evaluate the model. The model's final performance is assessed by calculating the mean of the test scores from each outer fold after all iterations of the outer loop have been completed. This average score reflects the anticipated performance of the model on unseen data when its hyperparameters are optimized.

References

1. Wainer, J., & Cawley, G. (2021). Nested cross-validation when selecting classifiers is overzealous for most practical applications. Expert Systems with Applications, 182, 115222.
2. Akiba, T., Sano, S., Yanase, T., Ohta, T., & Koyama, M. (2019). Optuna: A next-generation hyperparameter optimization framework. In Proceedings of the 25th ACM SIGKDD international conference on knowledge discovery & data mining, pp. 2623-2631.
3. Snoek, J., Larochelle, H., & Adams, R. P. (2012). Practical bayesian optimization of machine learning algorithms. Advances in neural information processing systems, 25.
4. Bergstra, J., Bardenet, R., Bengio, Y., & Kégl, B. (2011). Algorithms for hyper-parameter optimization. Advances in neural information processing systems, 24.
5. Prokhorenkova, L., Gusev, G., Vorobev, A., Dorogush, A. V., & Gulin, A. (2018). CatBoost: unbiased boosting with categorical features. Advances in neural information processing systems, 31.
6. CatBoost – Parameter tuning. [Online]. Available at: https://catboost.ai/en/docs/concepts/parameter-tuning
