# Supplementary material for: Real-Time Estimation of Arterial Partial Pressure of Carbon Dioxide in Patients Undergoing General Anesthesia: Predictive Modeling Study
Source: JMIR Med Inform. 2025 Sep 16;13:e64855. doi: 10.2196/64855 (PMC12439857; doi:10.2196/64855)
Supplement: Multimedia Appendix 4 [file medinform-v13-e64855-s004.pdf]

Multimedia appendix 4. Descriptive statistics of the selected variables

Characteristic	Missing	Total (n=4,651)	Subgroups Hypocapnic cases (n=179)	Normocapnic cases (n=3,328)	Hypercapnic cases (n=1,144)	p-Value
**Demographics**						
Age (median, IQR)	0	61.00 [52.00,69.00]	52.00 [40.50,60.00]	60.00 [51.00,68.00]	64.00 [56.00,71.00]	< 0.001
Gender (n, %)	0					< 0.001
Men		2,837 (61.00)	86 (48.04)	1,923 (57.78)	828 (72.38)	
Women		1,814 (39.00)	93 (51.96)	1,405 (42.22)	316 (27.62)	
Height* (median, IQR)	0	164.00 [157.50,169.95]	163.30 [156.30,169.20]	163.50 [157.00,169.80]	165.40 [159.00,170.00]	< 0.001
Weight* (median, IQR)	0	61.10 [53.48,69.10]	58.80 [51.62,66.80]	60.70 [53.25,68.30]	63.40 [54.70,71.75]	< 0.001
**Clinical information**						
Approach (n, %)	0					< 0.001
Open		2,333 (50.16)	119 (66.48)	1,781 (53.52)	433 (37.85)	
Videoscopic		1,998 (42.96)	43 (24.02)	1,346 (40.44)	609 (53.23)	
Robotic		320 (6.88)	17 (9.50)	201 (6.04)	102 (8.92)	
Optype (n, %)	0					< 0.001
Transplantation		733 (15.76)	28 (15.64)	531 (15.96)	174 (15.21)	
Major resection		822 (17.67)	22 (12.29)	527 (15.84)	273 (23.86)	
Minor resection		526 (11.31)	7 (3.91)	332 (9.98)	187 (16.35)	
Stomach		541 (11.63)	21 (11.73)	449 (13.49)	71 (6.21)	
Biliary/Pancreas		527 (11.33)	28 (15.64)	433 (13.01)	66 (5.77)	
Hepatic		371 (7.98)	17 (9.50)	246 (7.39)	108 (9.44)	
Colorectal		319 (6.86)	12 (6.70)	253 (7.60)	54 (4.72)	
Vascular		151 (3.25)	4 (2.23)	111 (3.34)	36 (3.15)	
Thyroid		52 (1.12)	6 (3.35)	41 (1.23)	5 (0.44)	
Breast		14 (0.30)	3 (1.68)	6 (0.18)	5 (0.44)	
Others		595 (12.79)	31 (17.32)	399 (11.99)	165 (14.42)	
PFT (n, %)	0					< 0.001
Normal		3,782 (81.32)	158 (88.27)	2,724 (81.85)	900 (78.67)	
Mild obstructive		367 (7.89)	5 (2.79)	239 (7.18)	123 (10.75)	
Mild restrictive		228 (4.90)	10 (5.59)	180 (5.41)	38 (3.32)	
Moderate obstructive		107 (2.30)	0 (0.00)	70 (2.10)	37 (3.23)	
Mixed or pure obstructive		108 (2.32)	1 (0.56)	72 (2.16)	35 (3.06)	
Moderate restrictive		42 (0.90)	3 (1.68)	31 (0.93)	8 (0.70)	
Severe restrictive		8 (0.17)	0 (0.00)	6 (0.18)	2 (0.17)	

Borderline obstructive		6 (0.13)	2 (1.12)	3 (0.09)	1 (0.09)	
Severe obstructive		3 (0.06)	0 (0.00)	3 (0.09)	0 (0.00)	
**Hemodynamic parameters**						
BT (median, IQR)	509	35.90 [35.50,36.20]	36.20 [35.70,36.50]	35.90 [35.50,36.30]	35.80 [35.40,36.10]	< 0.001
HR (median, IQR)	322	73.00 [64.00,84.00]	74.00 [64.00,86.50]	72.00 [63.00,83.00]	75.00 [66.00,86.00]	< 0.001
SPO_2 (median, IQR)	307	100.00 [100.00,100.00]	100.00 [100.00,100.00]	100.00 [100.00,100.00]	100.00 [99.00,100.00]	< 0.001
$ETCO_2$ (median, IQR)	297	35.00 [33.00,37.00]	32.00 [30.00,33.00]	34.00 [33.00,36.00]	37.00 [35.00,40.00]	< 0.001
MV (median, IQR)	573	5.40 [4.60,6.50]	5.60 [4.90,6.65]	5.40 [4.60,6.50]	5.40 [4.50,6.40]	0.065
MAWP (median, IQR)	319	8.00 [4.00,9.00]	8.00 [7.00,8.00]	8.00 [4.00,9.00]	8.00 [5.00,10.00]	< 0.001
RR (median, IQR)	324	14.00 [12.00,16.00]	13.00 [12.00,14.00]	14.00 [12.00,16.00]	16.00 [14.00,17.00]	< 0.001
PEEP* (median, IQR)	327	5.00 [0.00,5.00]	5.00 [4.00,5.00]	5.00 [0.00,5.00]	4.00 [0.00,5.00]	< 0.001
PIP* (median, IQR)	325	18.00 [14.00,22.00]	16.00 [14.00,19.00]	17.00 [14.00,21.00]	20.00 [16.00,24.00]	< 0.001
PPLAT* (median, IQR)	327	16.00 [13.00,20.00]	16.00 [13.00,18.00]	16.00 [13.00,19.00]	18.00 [14.00,21.00]	< 0.001
VT* (median, IQR)	322	390.00 [333.00,448.00]	449.00 [392.50,503.00]	397.50 [339.00,454.00]	355.00 [314.38,412.12]	< 0.001
FIO_2* (median, IQR)	319	40.00 [35.00,56.00]	36.00 [34.00,42.25]	37.00 [35.00,47.00]	47.00 [36.00,75.00]	< 0.001
**Constructed Features**						
CO (median, IQR)	386	5.54 [4.71,6.56]	5.81 [5.20,6.71]	5.47 [4.66,6.50]	5.72 [4.81,6.76]	< 0.001
VT/IBW (median, IQR)	322	6.51 [5.74,7.78]	8.08 [6.90,8.76]	6.74 [5.89,7.96]	5.92 [5.35,6.74]	< 0.001
SPO_2/FIO_2 (median, IQR)	329	2.50 [1.78,2.86]	2.78 [2.36,2.94]	2.70 [2.13,2.86]	2.13 [1.32,2.78]	< 0.001
$PEEP/FIO_2$ (median, IQR)	327	0.07 [0.00,0.12]	0.13 [0.06,0.15]	0.07 [0.00,0.14]	0.05 [0.00,0.09]	< 0.001
CRS (median, IQR)	327	30.35 [23.36,38.00]	39.68 [31.23,47.81]	31.54 [24.85,38.80]	25.34 [19.74,32.67]	< 0.001
RSBI (median, IQR)	325	27.00 [21.91,34.08]	36.47 [28.36,41.96]	28.33 [22.87,35.10]	23.22 [19.53,28.42]	< 0.001

IQR=Interquartile range; PFT=Preoperative pulmonary function test; BT=Body Temperature; HR=Heart rate; SPO_2=Percutaneous oxygen saturation; $ETCO_2$=End-tidal carbon dioxide; MV=Minute ventilation (from ventilator); MAWP=Mean airway pressure; RR=Respiratory rate based on capnography; PEEP=Positive and expiratory pressure; PIP=Peak inspiratory pressure; PPLAT=Plateau pressure; VT=Tidal volume; FIO_2=Fraction of inspired oxygen; CO=Estimated cardiac output; VT/IBW=Tidal volume per kilogram of ideal body weight; SPO_2/FIO_2=Oxygen saturation to fraction of inspired oxygen; $PEEP/FIO_2$=Positive end expiratory pressure to fraction of inspired oxygen; CRS=Compliance of the respiratory system; RSBI=Rapid shallow breathing index

* Used for establishing constructed features (not for model training)
